# Partial inhibition and bilevel optimization in flux balance analysis

**DOI:** 10.1186/1471-2105-14-344

**Published:** 2013-11-29

**Authors:** Giuseppe Facchetti, Claudio Altafini

**Affiliations:** 1 International School for Advanced Studies) Statistical and Biological Physics Dept. - Via Bonomea 265 - 34136, Trieste, Italy; 2 SISSA (International School for Advanced Studies) Functional Analysis Dept. - Via Bonomea 265 - 34136, Trieste, Italy

## Abstract

**Motivation:**

Within Flux Balance Analysis, the investigation of complex subtasks, such as finding the optimal perturbation of the network or finding an optimal combination of drugs, often requires to set up a bilevel optimization problem. In order to keep the linearity and convexity of these nested optimization problems, an ON/OFF description of the effect of the perturbation (i.e. Boolean variable) is normally used. This restriction may not be realistic when one wants, for instance, to describe the partial inhibition of a reaction induced by a drug.

**Results:**

In this paper we present a formulation of the bilevel optimization which overcomes the oversimplified ON/OFF modeling while preserving the linear nature of the problem. A case study is considered: the search of the best multi-drug treatment which modulates an objective reaction and has the minimal perturbation on the whole network. The drug inhibition is described and modulated through a convex combination of a fixed number of Boolean variables. The results obtained from the application of the algorithm to the core metabolism of *E.coli* highlight the possibility of finding a broader spectrum of drug combinations compared to a simple ON/OFF modeling.

**Conclusions:**

The method we have presented is capable of treating partial inhibition inside a bilevel optimization, without loosing the linearity property, and with reasonable computational performances also on large metabolic networks. The more fine-graded representation of the perturbation allows to enlarge the repertoire of synergistic combination of drugs for tasks such as selective perturbation of cellular metabolism. This may encourage the use of the approach also for other cases in which a more realistic modeling is required.

## Background

In recent years, genome-scale metabolic networks have represented an important paradigm of systems biology, well describing how interesting (and relevant) biological features can be deduced in spite of the complexity of the model [1-4]. Thanks to the use of genomic techniques, metabolic networks have been reconstructed for many organisms, ranging from small bacteria up to the human cells. In parallel, the development of quantitative descriptions of these large and complex systems based on simple computational framework such as Flux Balance Analysis (FBA) [5,6] has increased both their characterization [7-9] and the spectrum of applications. Two important examples are (i) strain improvement [10,11], i.e. the identification of the best knockout or gene manipulation maximizing the biosynthesis of a key metabolite, (ii) support to drug discovery through the identification of new inhibition targets [12-15] or of new drug therapies for various medical purposes [16,17]. All the studies just mentioned are based on the FBA formalism.

FBA is a linear constraint-based framework for stoichiometric models of metabolic networks; the network is described by the stoichiometric matrix **S** = (*s*_*i*,*j*_), where *s*_*i*,*j*_ represents the stoichiometric coefficient of the *i*-th metabolite in the *j*-th reaction (with *i* = 1,…,*m*, *j* = 1,…,*r*), and by the reaction fluxes denoted by the vector $v\in R^{r}$ (including chemical transformations, transports, nutrients supply and waste disposal processes). Because of the much faster dynamics compared to gene regulation, metabolic processes are assumed to be at steady state, which corresponds to imposing

(1) Sv=0

(this holds for all the metabolites since the vector **v** includes all the processes). Thermodynamical constraints and availability of nutrients add further constraints, such as finite lower (*L*_*i*_) and upper-bounds (*U*_*i*_) on the fluxes:

(2) $$L_{i}\leq v_{i}\leq U_{i}\;\forall i=1,\ldots ,r.$$

The constraints (1) and (2) generate a convex and bounded set $W\subset R^{r}$ to which the vector **v** has to belong. To obtain the reaction fluxes (a point in *W*) which describe the metabolic state of the organism, one has to perform the maximization of a function **Φ**(**v**) (or equivalently the minimization of -**Φ**(**v**)). The choice of **Φ**(**v**) depends on the context and on the application: common examples are biomass production [18], ATP production [2] and minimal metabolic adjustment [19].

When applications like strain engineering or drug target identification are treated with this formalism, an additional function, **Ψ**[**v**(**h**)], is normally introduced, where **h** are the variables we can control and which describe, for example, the knockouts or the drug inhibitions inducing a reduction of the set *W* to *W*(**h**) (see later for its more precise definition). The corresponding secondary optimization problem can consist, for instance, of the maximization of the production of a certain metabolite [10] or of the maximal inhibition of a target reaction [16]. The problem becomes therefore a bilevel optimization [20]:

(3) $$\begin{array}{cc} \text{arg max} & \Psi \left[v(h)\right].\\ \begin{array}{l} h:\left[\begin{array}{c} v(h)\in \text{arg min}\Phi (w)\\ \;w\in W(h) \end{array}\right] \end{array} & \end{array}$$

where arg min_*x*∈*Q*_*f*(*x*) stands for the set of *x* for which the function *f* attains its minimum value in *Q* (or equivalently, its maximum value for "arg max"). Therefore, (3) says that the output of the bilevel optimization is the vector **h** such that the corresponding vector **v**(**h**), which minimizes **Φ** on *W*(**h**), maximizes the function **Ψ**. The reformulation of a bilevel optimization as a single optimization is commonly obtained through duality theory [21,22]. When performing this reformulation, in order to save the linear nature of the optimization procedure, the variables **h** (the real output of the algorithm) have to be Boolean, rather than continuous [2,10,13]. While this approach is correct for gene knock-out, in the case of drug treatment (where the enzymes are inhibited by drug) or gene modification (on which one changes the enzymes activity) it represents only a rough approximation which may not constitute a realistic description of the biological effect. It is in fact more plausible to assume that a drug acting on an enzyme leads to a partial loss of functionality of the latter, and hence to a partial inhibition of the corresponding reaction(s). In order to treat the inhibition as a variable to be optimized and to avoid the ON/OFF oversimplification it is necessary to reformulate the bilevel optimization as a nonlinear (nonconvex) single optimization problem [23] but this leads to a more complicated situation from a numerical point of view.

Although a complete inhibition of a disease-causing target may not represent the right therapeutic solution (in healthy cells, the level of each metabolite must be in a finite range) and although expected to be a potential strategy in a multi-target approach [24], partial inhibition has been considered only in a few computational works; moreover, studies like [25,26] dealt with a small part of the network, modeling the kinetic reactions explicitly and solving then numerically. The partial inhibition then amounts, for instance, to a modulation of one or more kinetic parameters. Because of the complexity of the metabolic networks and because of the impossibility of knowing the kinetic parameters of all biochemical reactions, this approach cannot be applied at a genome-wide level. On the other hand, the authors of [27] consider the whole network, but the (partial) inhibition is given as initial fixed parameter of the model and only the effect of the perturbation is quantified. A different approach is presented in [28] in the context of the prediction of new drug targets; these targets are identified though a two-stages FBA (which differs from a bilevel formulation because the two optimizations are not nested). However, the potential targets obtained with this method must be verified exhaustively, which may represent a problem for networks with more than the 26 reactions of the human hyperuricemia metabolic pathway considered in [28].

Therefore, the aim of the present paper is to describe a novel algorithm which allows to provide a more realistic description of the partial inhibition induced by the drugs on large networks while still remaining within the framework of Linear Programming (LP). In order to introduce the algorithm, we refer to a realistic case where a bilevel minimization is used. Namely we consider the search for the optimal combination of drugs capable, through a synergistic effect, to inhibit (or enhance) an objective reaction (i.e. a putative target for a disease) while inducing the minimal perturbation on the rest of the network. Indeed, the selectivity of the therapy is one of the most important aspects of any drug discovery project. Replacing a single Boolean variable by a convex combination of a fixed number of Boolean variables, we are able to model the inhibition as any number belonging to a discretized representation of the interval [0,1]. This approach preserves the linear nature of the final problem. Notice that the method we propose can be extended to any bilevel optimization which needs to deviate from the simple ON/OFF description.

The paper is organized as follows. We first formalize our example about drug combinations; then, within this case study, we describe why Boolean variables are necessary in the reformulation of a bilevel optimization problem via the strong duality theorem of LP. The presentation of the basic idea of the proposed algorithm and the discussion of its limits conclude the Methods Section. Results and Discussion sections present and comment the outcomes obtained on a benchmark application to the *E.coli* core metabolism and to some other larger networks. Final considerations are then reported in the Conclusion.

## Methods

### Optimal drug combination: a guiding example

In FBA the vector **v** of the metabolic fluxes is obtained through the optimization of a certain function **Φ**(**v**). For unperturbed networks, the production of the macromolecular building blocks for the biomass (the growth rate) is often maximized [18]: we denote by **v**^ut^ (ut="untreated"; all symbols and variables are listed in Table 1) the reaction fluxes obtained after this optimization. This fluxes can be nonunique [29]: an analysis of the case in which **v**^ut^ has degenerate values is reported in the Additional file 1. In any case, throughout the paper these unperturbed fluxes are considered as given parameters of the problem. In the following all reactions are irreversible (*L*_*i*_ = 0, ∀*i* = 1,…,*r*): indeed, by decomposing any reversible reaction in a couple of irreversible reactions, we can always assume that fluxes have non-negative values.

**Table 1:** Symbol, value range, meaning and type of all quantities used in the algorithm description

***Network and drugs***			
**Symbol**	**Range**	**Description**	**Type**
*r*		Number of reactions	Parameter
*m*		Number of metabolites	Parameter
*d*		Number of drugs	Parameter
**S**	$R^{m\times r}$	Stoichiometric matrix of the network	Parameter
$v_{1}^{\text{ut}},\ldots ,v_{r}^{\text{ut}}$	$R_{+}$	Reaction fluxes of the untreated network	Parameter
$v_{1}^{\text{tr}},\ldots ,v_{r}^{\text{tr}}$	$R_{+}$	Reaction fluxes after drug treatment **h**	Variable
mod	1,…,*r*	Index of the reaction which must be modulated	Parameter
*W*	-	Set of feasible solutions for unperturbed network	Parameter
*W*(**h**)	-	Set of feasible solutions for network perturbed by drug inhibition **h**	Parameter(*)
	-	Set of drugs ( |D|=d)	Parameter
$T_{k}$	-	Set of targets of drug *k* (*k* = 1,…,*d*)	Parameter
***Outer problem***			
**Symbol**	**Range**	**Description**	**Type**
*τ*	(0,1)	Threshold for the reaction that must be inhibited ( $v_{\text{mod}}^{\text{tr}}\leq \tau v_{\text{mod}}^{\text{ut}}$)	Parameter
	>1	Threshold for the reaction that must be activated ( $v_{\text{mod}}^{\text{tr}}\geq \tau v_{\text{mod}}^{\text{ut}}$)	
*P*	$N_{0}$	Precision parameter	Parameter
*h*_1_,…,*h*_*d*_	[0,1]	Drug inhibitions	Variable
*x*_1,0_,*x*_1,1_,…,*x*_*d*,*P*_	{0,1}	Drug-dosage variables	Variable
*b*	10^-3^	Correction against an "overselection" of drugs	Parameter
***Inner problem***			
**Symbol**	**Range**	**Description**	**Type**
*v*_1_,…,*v*_*r*_	$R_{+}$	Reaction fluxes (primal problem and outer problem)	Variable
*L*_1_,…,*L*_*r*_	$R_{+}$	Lower-bounds for the reaction fluxes (of the primal problem)	Parameter
*U*_1_,…,*U*_*r*_	$R_{+}$	Upper-bounds for the reaction fluxes (of the primal problem)	Parameter
*a*_1_,…,*a*_*r*_	$R_{+}$	Absolute fluxes differences $a_{i}=|v_{i}-v_{i}^{\text{ut}}|$ (primal problem)	Variable
*μ*_1_,…,*μ*_*m*_		Dual variables associated to the FBA steady state constraint	Variable
*λ*_1_,…,*λ*_*r*_	$R_{+}$	Dual variables associated to the unperturbed upper-bounds	Variable
*δ*_1_,…,*δ*_*t*_	$R_{+}$	Dual variables associated to the drug inhibition; $t:=\sum_{k=1}^{d}T_{k}$	Variable
$\delta_{1}^{\text{max}},\ldots ,\delta_{t}^{\text{max}}$	$R_{+}$	Upper-bounds of the dual variables *δ*’s	Variable
*α*_1_,…,*α*_*r*_	$R_{+}$	Dual variables associated to the first absolute value inequalities	Variable
*β*_1_,…,*β*_*r*_	$R_{+}$	Dual variables associated to the second absolute value inequalities	Variable

(*): The set *W*(**h**) is considered as parameter in the sense that its dependence on the variables **h** is given.

The guiding example we introduce here consists in the search of the most selective combination of drugs: in particular, we suppose to have a metabolic network and a set of drugs which inhibit some reactions of this network (the set of targets of the *k*-th drug is indicated by $T_{k}$). We want to modulate a certain reaction (for example rendering its flux less than a given threshold) through a combination of these drugs, inducing the minimal effect on the rest of the network. In order to give a more clear presentation of the algorithm, we assume that a drug induces an identical fractional inhibition on all its targets. Therefore, the amount of inhibition by the *k*-th drug on its target reaction $i\in T_{k}$, can be modeled by the linear constraint

(4) $$v_{i}\leq U_{i}(1-h_{k}),$$

where *U*_*i*_ is the upper-bound of the flux *v*_*i*_ and where, for modeling with partial inhibition, *h*_*k*_ ∈ [0,1]. Through this formalism we do not consider the allosteric interaction between two (or more) drugs on the same enzyme: indeed, our model simply takes the maximum inhibition over the set of drugs which affect the enzyme of reaction *i*:

$$v_{i}\leq U_{i}(1-\underset{k:i\in T_{k}}{\text{max}}h_{k}).$$

Then, the vector **h** ∈ [0,1]^*d*^ represents the drug treatment, i.e. the inhibition due to the drugs: for example, for *d* = 3, the vector **h** = [0.5, 0, 0.8] indicates that drug 2 is not used (*h*_2_ = 0) while drugs 1 and 3 are used at dosages which cause respectively a 50% and 80% inhibition of their targets (hence, in (4), the reduction of their upper-bounds to 50% and 20% in the original values). For each choice of **h** these inhibitions reduce the set *W* (generated by the constraints (1) and (2)) to a subset *W*(**h**):

$$\begin{array}{ll} \;W(h)= & \{v\in W\;\text{such that}\;v_{i}\leq U_{i}(1-h_{k}),\\ & \;\;\forall k=1,\ldots ,d,\;\forall i\in T_{k}\}. \end{array}$$

The determination of the reaction fluxes **v**^tr^(**h**) (tr="treated") for the drug-treated network is obtained through the MOMA problem (Minimization Of Metabolic Adjustment) which has been shown to generate reasonable and realistic results for perturbed metabolism [4,19,30-32]. In order to apply the theory of linear programming, we use the definition of MOMA in terms of norm *L*^1^[33]. Then

(5) $$\begin{array}{ll} \;v^{\text{tr}}(h)= & \text{arg min}\;∥v-v^{\text{ut}}{∥}_{1}.\\ & v\in W(h) \end{array}$$

In the following the side effect of a drug treatment is quantified in terms of the distance ∥**v**^tr^(**h**) - **v**^ut^∥_1_ used in (5): the greater the distance, the bigger the impact of the drugs on the whole network.

The problem can be stated as follows:

Given:

• *a metabolic network, which means a stoichiometric matrix *$\text{S}\in R^{m\times r}$* and the upper-bounds*$U\in R^{r}$*of the reaction fluxes***v**;

• *the unperturbed fluxes***v**^ut^;

• *the set**of drugs together with their inhibition targets *${\left\{T_{k}\right\}}_{k=1,\ldots ,|D|}$*,*

• *the index (denoted by* "mod"*) of the objective reaction whose flux* (*v*_mod_) *must be modulated*;

• *a threshold* *τ* ∈ [0,1) *for the modulation constraint on v*_mod_;

*we want to find the inhibition***h** ∈ [0,1]^*d*^ *such that*$v_{\text{mod}}^{\text{tr}}(h)\leq \tau v_{\text{mod}}^{\text{ut}}$*and such that it causes the minimal side effect (i.e. the minimal distance* ∥**v**^tr^(**h**)-**v**^ut^∥_1_*).* Of course, a different definition of side effect as well as a different constraint on $v_{\text{mod}}^{\text{tr}}$ (perhaps on its maximal value [17]) can be used if needed by the problem.

According to (5), for a given set of drugs (i.e., for a given inhibition vector **h**), we can calculate both **v**^tr^(**h**) (and then check whether $v_{\text{mod}}^{\text{tr}}(h)\leq \tau v_{\text{mod}}^{\text{ut}}$) and the value of the side effect. Similarly to (3), the final formulation of the problem is the following:

(6) $$\begin{array}{cc} \min & ∥v^{\text{tr}}(h)-v^{\text{ut}}{∥}_{1}.\\ \begin{array}{l} h:\left[\begin{array}{l} v^{\text{tr}}(h)=\text{arg min}∥\text{w}-v^{\text{ut}}{∥}_{1}\\ \;w\in W(h)\\ v_{\text{mod}}^{\text{tr}}(h)\leq \tau v_{\text{mod}}^{\text{ut}} \end{array}\right] \end{array} & \end{array}$$

The bilevel optimization (6) is a min-min linear program. The inner problem adjusts the fluxes so as to achieve the minimal metabolic adjustment, subject to the drug inhibitions imposed by the outer problem and to the stoichiometric constraints. The outer problem selects the combination of drugs which has the minimum side effect and guarantees a modulated flux lower than the desired threshold.

Since we are looking for a minimum, the absolute value operation $a_{i}=|v_{i}-v_{i}^{\text{ut}}|$, necessary for the definition of the *L*^1^-norm, is reformulated in terms of the following linear inequalities:

$$\begin{array}{l} a_{i}\geq +(v_{i}-v_{i}^{\text{ut}});\\ a_{i}\geq -(v_{i}-v_{i}^{\text{ut}}). \end{array}$$

The sum of *a*_*i*_ (i.e. $\sum_{i=1}^{r}a_{i}=\sum_{i=1}^{r}|v_{i}-v_{i}^{\text{ut}}|=∥v^{\text{tr}}(h)-v^{\text{ut}}{∥}_{1}$) defines both the objective function of the inner and the outer problem. In fact in (6), at the optimal point of the inner problem (at the minimum of ∥**w** - **v**^ut^∥_1_) we have that **w** = **v**^tr^(**h**), hence ∥**w** - **v**^ut^∥_1_ is equal to the objective function of the outer problem. Notice that, despite of the common objective function, the two minimization cannot be merged in a single optimization because of the additional constraint on *v*_mod_ contained in the outer problem. Indeed, calling *B* the set defined by the inequality $v_{\text{mod}}^{\text{tr}}(h)\leq \tau v_{\text{mod}}^{\text{ut}}$, the following relation holds:

$$\left\{\begin{array}{cc} \text{arg min} & f(v)\\ v\in W(h) & \end{array}\right\}\cap B\neq \left\{\begin{array}{cc} \text{arg min} & f(v)\\ v\in W(h)\cap B & \end{array}\right\}.$$

Then, the detailed equations of the bilevel optimization (6) are the following:

$$\begin{array}{ll} \text{Minimize} & \sum_{i=1}^{r}a_{i}\;\text{"outer problem"}\\ \text{such that} & \\ & \left[\begin{array}{lll} \text{Minimize} & \sum_{i=1}^{r}a_{i}\;\text{"inner problem"} & \\ \text{such that} & & \\ & \sum_{j=1}^{r}S_{i,j}v_{j}=0 & \\ & v_{i}\leq U_{i} & \\ & v_{i}\leq U_{i}(1-h_{k}) & \\ & +\;v_{i}-a_{i}\leq +\;v_{i}^{\text{ut}} & \\ & -\;v_{i}-a_{i}\leq -\;v_{i}^{\text{ut}} & \end{array}\right]\\ & v_{\text{mod}}\leq \tau v_{\text{mod}}^{\text{ut}}, \end{array}$$

### The strong duality theorem and the need of Boolean variables

This bilevel optimization is commonly solved by applying the strong duality theorem of LP [10,11,34] which states: *"Let A be a matrix, and let***b*** and***c*** be vectors. Then*

$$\begin{array}{l} \max\{c^{T}x\;\text{such that}\;Ax\leq b\;,\;x\geq 0\}\\ \;=\min\{b^{T}\theta \;\text{such that}\;A^{T}\theta \geq c\}, \end{array}$$

*providing that both sets are not empty"*[22], where **x** are the primal variables and *θ* are the dual variables (note that, because of the use of the transpose of the matrix *A* in the dual problem, there is a dual variable for each constraint of the primal problem). Therefore, the application of this theorem to the inner problem consists in appending a list of constraints, *A*^*T*^ *θ* ≥ **c**, corresponding to the dual form of the constraints of the inner problem and setting the inner objective function equal to its dual **c**^*T*^**x** = **b**^*T*^*θ* (see Additional file 2 for a depiction of the structure of the matrix eventually obtained). Since, from the strong duality theorem, this equality holds only at the optimal points of both primal and dual problems, the resulting set of constraints is equivalent to selecting only the solutions of the inner problem.

This leads to the following single minimization, in which Greek letters refer to dual variables (for clarity, we differentiate them according to the associated constraints of the primal problem, as detailed in Table 1):

(7a) $$\begin{array}{l} \text{Minimize}\;\;\overset{r}{\underset{i=1}{\sum }}a_{i}\;\;\text{such that}\; \end{array}$$

(7b) $$\begin{array}{l} \overset{r}{\underset{j=1}{\sum }}S_{i,j}v_{j}=0\;\;\forall i=1,\ldots ,m;\; \end{array}$$

(7c) $$\begin{array}{l} v_{i}\leq U_{i}\;\;\forall i=1,\ldots ,r;\; \end{array}$$

(7d) $$\begin{array}{l} v_{i}\leq U_{i}(1-h_{k})\;\;\forall k=1,\ldots ,d,\;i\in T_{k};\; \end{array}$$

(7e) $$\begin{array}{l} v_{i}-a_{i}\leq +v_{i}^{\text{ut}}\;\;\forall i=1,\ldots ,r;\; \end{array}$$

(7f) $$\begin{array}{l} -v_{i}-a_{i}\leq -v_{i}^{\text{ut}}\;\;\forall i=1,\ldots ,r;\; \end{array}$$

(7g) $$\begin{array}{l} \overset{m}{\underset{i=1}{\sum }}S_{i,j}\mu_{i}+\lambda_{j}+\underset{i:j\in T_{i}}{\sum }\delta_{i}+\alpha_{j}-\beta_{j}\geq 0\;\;\forall j=1,\ldots ,r;\; \end{array}$$

(7h) $$\begin{array}{l} \alpha_{j}+\beta_{j}\leq 1\;\;\forall j=1,\ldots ,r;\; \end{array}$$

(7i) $$\begin{array}{l} v_{\text{mod}}\leq \tau v_{\text{mod}}^{\text{ut}};\; \end{array}$$

(7j) $$\begin{array}{l} -\overset{r}{\underset{i=1}{\sum }}a_{i}=\overset{r}{\underset{i=1}{\sum }}\left(\lambda_{i}+U_{i}\delta_{i}\underset{k:i\in T_{k}}{\sum }(1-h_{k})+(\alpha_{i}-\beta_{i})v_{i}^{\text{ut}}\right),\; \end{array}$$

where (7a) specifies the objective function of the outer problem; equations (7b)–(7f) refer to the primal inner problem (the constraints of the original inner problem); (7g) and (7h) are the dual constraints; (7i) imposes the outer problem contraint on *v*_mod_ and (7j) is the duality theorem equality (namely **c**^*T*^**x** = **b**^*T*^*θ*). However, this last equation is no longer a linear constraint since it contains the product between the outer problem variable *h*_*k*_ and the dual variable *δ*_*i*_; hence, the problem can no longer be solved by a linear optimization. It is common to overcome this complication by restricting the *h*_*k*_ variables to Boolean values. In this case, in fact, the nonlinear terms *δ*_*i*_*h*_*k*_ can be exactly linearized as follows:

(8) $$\begin{array}{c} z_{i,k}:=\delta_{i}h_{k};\\ 0\leq z_{i,k}\leq \delta_{i}^{\text{max}}h_{k};\\ \delta_{i}-\delta_{i}^{\text{max}}(1-h_{k})\leq z_{i,k}\leq \delta_{i}, \end{array}$$

where $\delta_{i}^{\text{max}}$ is the upper bound for the dual variable *δ*_*i*_.

The restriction to Boolean variables saves the linear nature of the problem (which however requires now Mixed Integer Linear Programming) but it implies the assumption that drugs can only act as switches on the reactions, or equivalently, that we are considering only an ON/OFF model.

### Partial inhibition

In this Section we propose a solution which can still use the duality theorem for solving the bilevel optimization while including the possibility of inducing a partial inhibition of the reactions targeted by the drugs. This requires to create a discretization of the interval [0,1] and to replace the ON/OFF action of each drug with *P* + 1 Boolean variables describing this discretization (*P* is a fixed parameter of the problem). For the *k*-th drug (*k* = 1,…,*d*) we introduce the set of Boolean variables {*x*_*k*,*n*_}_*n*=0,…,*P*_ and define an inhibition coefficient *h*_*k*_ by the following convex combination:

(9) $$h_{k}:=\frac{x_{k,0}}{2^{P}}+\overset{P}{\underset{n=1}{\sum }}\frac{x_{k,n}}{2^{n}}.$$

In (9) the integer *P* is related to the desired accuracy of the [0,1] discretization. Indeed, the factor *h*_*k*_ assumes values between 0 and 1 with precision 2^-*P*^. Notice that for *P* = 0 we have the ON/OFF model of the previous section. We can replace now (4) with the following inequality:

$$v_{i}\leq U_{i}(1-h_{k})=U_{i}\left(1-\frac{x_{k,0}}{2^{P}}-\overset{P}{\underset{n=1}{\sum }}\frac{x_{k,n}}{2^{n}}\right).$$

When the strong duality theorem is applied, the nonlinear terms are the *δ*_*i*_*h*_*k*_ products. Expanding the product according to the definition in Eq. (9):

$$\delta_{i}h_{k}=\frac{\delta_{i}x_{k,0}}{2^{P}}+\overset{P}{\underset{n=1}{\sum }}\frac{\delta_{i}x_{k,n}}{2^{n}},$$

the nonlinearity is now spread over the products *δ*_*i*_*x*_*k*,*n*_ with again *x*_*k*,*n*_ a Boolean variables. Similarly to (8), we can write an equivalent set of linear inequalities:

$$\begin{array}{c} z_{i,k,n}:=\delta_{i}x_{k,n};\\ 0\leq z_{i,k,n}\leq \delta_{i}^{\text{max}}x_{k,n};\\ \delta_{i}-\delta_{i}^{\text{max}}(1-x_{k,n})\leq z_{i,k,n}\leq \delta_{i}. \end{array}$$

**Figure 1:**
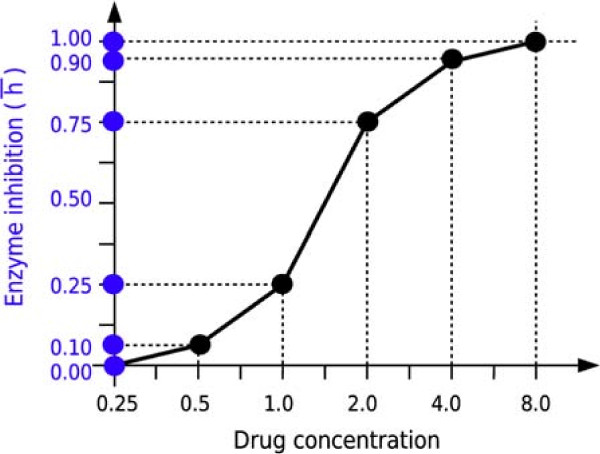
**Constructing the inhibition** ***h* from the experimental dose-response curve: an example.** The points of the curve are hypothetical experimental measurements of the effect of the drug on the activity of the enzyme. The discretization of the curve can be used as basis for the discretization of the interval [0,1]: therefore, referring to (10), we may define *h*_*k*_ = 0.10*x*_*k*,0_ + 0.15*x*_*k*,1_ + 0.50*x*_*k*,2_ + 0.15*x*_*k*,3_ + 0.10*x*_*k*,4_.

Notice that any "representation" of partial inhibition values can be used in place of (9). Let us imagine, for instance, that we would like *h*_*k*_ to have the same values obtained in the dose-response experiments for the determination of the half maximal inhibitory concentration (IC_50_) of drug *k* (see Figure 1):

$$h_{k}\in \{0,\;{\bar{h}}_{k,0},\;{\bar{h}}_{k,1},\;\ldots ,\;{\bar{h}}_{k,P},\;1\};$$

(with $0<{\bar{h}}_{k,i}<{\bar{h}}_{k,j}<1,\forall i<j$) then we may define *h*_*k*_ by the following convex combination

(10) $$h_{k}:={\bar{h}}_{k,0}x_{k,0}+({\bar{h}}_{k,1}-{\bar{h}}_{k,0})x_{k,1}+\cdots +(1-{\bar{h}}_{k,P})x_{k,P},$$

with a series of inequalities

$$x_{k,0}\leq x_{k,1}\leq x_{k,2}\cdots \leq x_{k,P}.$$

Of course, the discretization (9) is the most efficient because, for a given number of Boolean variables (*P* + 1), it generates the maximum precision (2^-*P*^). For this reason, in the following we will refer to (9) only.

### Inhibitions and activation of the objective reaction

The evaluation of the effect on the fluxes induced by the drugs is performed through the MOMA formalism. It is known that this approach describes well the spreading across the network of the effect of the perturbation: many processes are down-regulated or up-regulated in order to adjust and compensate the effect of the perturbation (see for example [4]). For the same reason, a second intervention may amplify the deactivation (recovery) of a certain metabolic function that was down-regulated (activated) after the first perturbation [30]. In terms of multiple drug effect, this means that a drug synergism may reinforce both the inhibition and the activation of the reaction fluxes.

In our algorithm, one can selects between these two situations through a different constraint on *v*_mod_ in the outer problem: indeed, imposing as in the previous Section

$$v_{\text{mod}}\leq \tau v_{\text{mod}}^{\text{ut}},$$

(for 0 ≤ *τ* < 1) the algorithm identifies synergistic inhibitions, whereas requiring

$$v_{\text{mod}}\geq \tau v_{\text{mod}}^{\text{ut}},$$

(for *τ* > 1) the algorithm generates drug interactions that up-regulates the objective reaction.

In the following, both versions are applied.

### Cases of multiple equivalent solutions (non-uniqueness)

**Figure 2:**
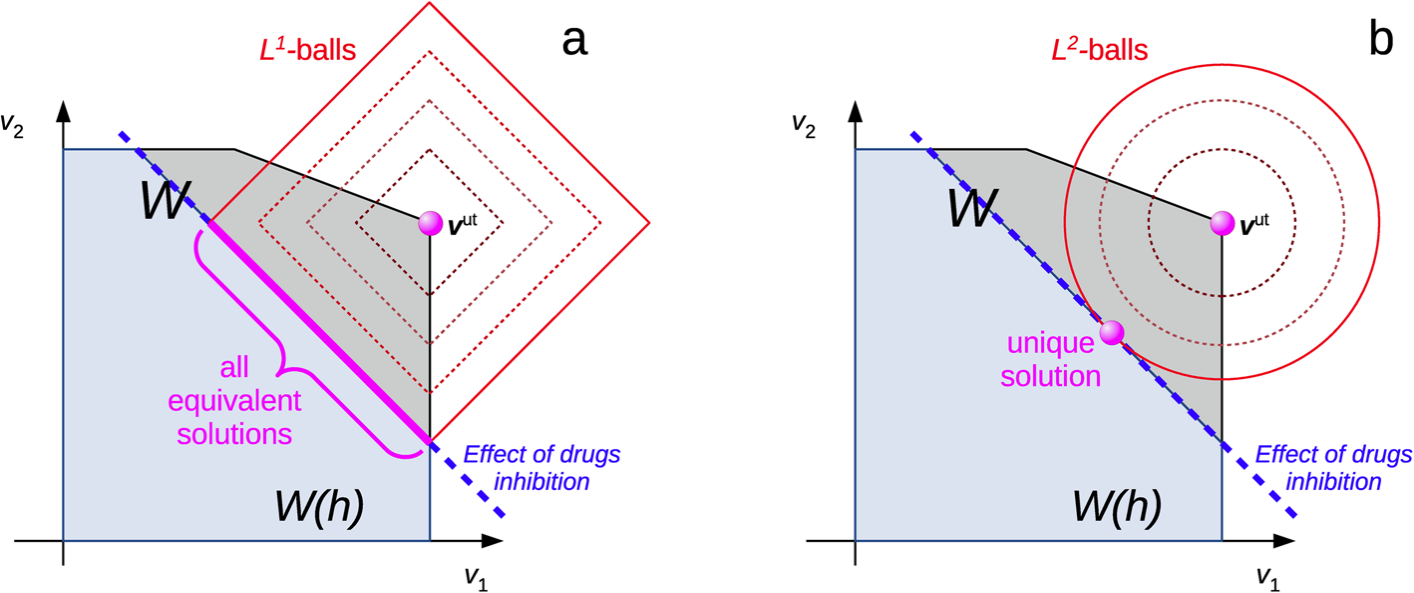
**Convexity of** ***L***^**1**^**- and** ***L***^**2**^**-formulations and uniqueness of the solution.** The two pictures report the unperturbed fluxes (**v**^ut^) in the original set *W*. When the inhibition *h* is applied (dotted blue line), the set reduces to *W*(*h*). **(a)** With the *L*^1^-norm, balls are not strictly convex, therefore cases of multiple equivalent solutions may appear. As one can see, these situations occur only when the hyperplane of *W*(**h**) which realizes the minimum distance with respect to **v**^ut^ is parallel to an edge of the *L*^1^-ball. **(b)** Conversely, *L*^2^ balls are strictly convex and always lead to a unique solution.

Apart from the nonuniqueness of the unpertubed fluxes **v**^ut^ analyzed in the Additional file 1, there are also other cases of degeneracy of the outcome of the algorithm, which we present here. Indeed, it is worth noting that the use of the norm *L*^1^ does not guarantee the uniqueness of the solution: indeed balls in *L*^1^ and the polytope *W*(**h**) are convex but not strictly convex sets. Unfortunately this limit can be overcome only passing to the *L*^2^ formulation with the consequent loss of the linearity of the problem. However, we expect that such a type of situations are quite rare since they appear only when the hyperplane (or the intersection of some of them) which defines *W*(**h**) and which realizes the minimum distance with respect to the vector **v**^ut^, is parallel to an edge (or face) of the *L*^1^-ball (see Figure 2).

Other (more common) cases of multiple equivalent solutions (i.e. solutions with the same network perturbation ∥**v**^ut^-**v**^tr^∥_1_) are avoided through a specific correction mechanism. For instance, if there exists a pair of drugs *k* and *l* such that $T_{k}\subset T_{l}$ (i.e. drug *k* inhibits enzymes which are already target by drug *l*), any solution which contains both drugs is equivalent to the solution without drug *k* (i.e. drug *k* is superfluous). Similar reasoning can be done between a lower and higher dosages of the same drug. Therefore, in order to prevent an "overselection" of drugs, we introduce an additional term in the objective function of the outer problem. The new function becomes the following:

$$\overset{r}{\underset{i=1}{\sum }}a_{i}+b\overset{d}{\underset{k=1}{\sum }}h_{k},$$

where the parameter *b* is chosen small enough (10^-3^ in our computations, much smaller than 10^3^, the common upper-bounds of the fluxes) in order to keep this term smaller than the difference in the side effects and therefore not to change the order between non-equivalent solutions.

A similar approach is used to solve the redundancy in the definition of *h*_*k*_. Indeed, since in (9) *x*_*k*,0_ and *x*_*k*,1_ have the same coefficient, the inhibition *h*_*k*_ does not change when swapping the values of these two variables. To avoid this degeneracy, we require that *x*_*k*,0_ be one only if all the other *x*_*k*,*j*_ (for *j* = 1,…,*P*) are one. This is obtained through the following extra linear constraint:

$$x_{k,0}\leq \frac{1}{P}\overset{P}{\underset{j=1}{\sum }}x_{k,j}.$$

Since the problem is not strictly convex and since equivalent drug combinations may always appear (for instance, the combination of drug A which targets reaction 1 plus drug B which targets reactions 2 and 3 is equivalent to the combination of drug C which targets reaction 2 plus drug D which targets reactions 1 and 3), the problem of multiple equivalent solutions needs to be considered. In all these cases, because of the numerical implementation of the algorithm, a random choice of one of these optimal solutions is taken. Since, by definition, all these equivalent solutions fulfill the conditions on the objective reaction and on the minimization of the side effect, their differences are irrelevant: indeed they concern only some other fluxes on which we do not have any specific requirements. For this reason any solution chosen by the implementation of the algorithm can be considered acceptable.

## Results

### Computational performaces

The proposed algorithm has been implemented in MATLAB (2012R) and the optimization has been performed using ILOG-IBM CPLEX 12.1 (http://www-01.ibm.com/software/commerce/optimization/cplex-optimizer/) under academic license.

**Figure 3:**
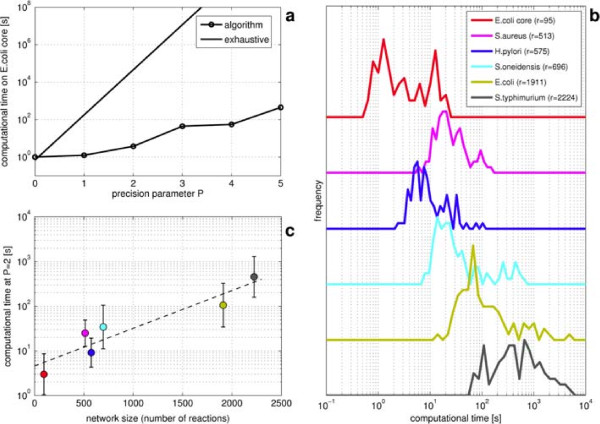
**Algorithm performances.** **(a)** Computational time of the algorithm compared with an estimation of the exhaustive search for different values of *P* on the *E.coli* core metabolism. **(b)** The influence of the size of the network on the performances is here reported as distribution of the computational time for the 6 metabolic networks listed in Table 2. Each distribution is built over 100 random samples obtained by changing the targets of a fixed number of drugs and keeping the precision parameter and the threshold constant (*P* = 2 and *τ* = 0.35). **(c)**: Averaged computational time (same color code of the corresponding distributions reported in panel (b)) and error bar as a function of the size of the network (expressed as number of reactions, see Table 2). All tests have been carried out using the software ILOG-IBM CPLEX on a 2.3 GHz CPU.

First, the impact of the parameter *P* on the computational cost is evaluated on the core metabolism of *E.coli* (see Table 2 for the main features of the corresponding network and the number of drugs that have been selected from online databases): we choose *ribose-5-phosphate isomerase* as reaction to be modulated (*τ* = 0.35) and run the algorithm with different values of *P* from 0 to 5, recording the computational time required to find the solution. Since there are 8 drugs, the extremal values of *P* correspond to 8 and 48 Boolean variables in the whole problem. Notice that for *P* = 5, the accuracy on the definition of *h*_*k*_ is quite high (2^-*P*^ = 1/32 < 5*%*). In addition, we estimate the time needed to perform the evaluation of the inhibitory effect (MOMA) of a single drug combination as an average over 20 random subsets of the 8 available drugs. From this value we can predict the approximate computational cost of an exhaustive search over all possible drug combinations (and dosages). The comparison of the performaces of the algorithm with this estimation is plotted in panel (a) of Figure 3.

**Table 2:** **Main properties of the metabolic networks used in this paper (from BIGG **http://bigg.ucsd.edu/**)**

	***E.coli* core**	***S.aureus***	***H.pylori***	**S.oneidensis**	***E.coli***	***S.typhimurium***
Number of reactions	95	575	513	696	1911	2224
Number of metabolites	72	455	436	528	1337	1497
Number of genes	137	619	339	783	1261	1271
Number of drugs	8	-	-	-	-	-
Reference	[35]	[36]	[37]	[38]	[39]	[40]

For the *E.coli* core network, drugs and respective targets have been selected from DrugBank database [41]: drugs which are common metabolites (for example Adenosine) have been discarded and drugs with the same targets have been grouped.

Moreover, we run the algorithm on the metabolic network of the six micro-organisms listed in Table 2. Our scope is to evaluate the impact of the size of the network (parametrized by the number of reactions *r*) on the computational performaces. In order to limit the interference of other parameters, these calculations are carried out with the same objective reaction (in particular we still keep *ribose-5-phosphate isomerase* since it appears on all networks we have considered) at constant precision (*P* = 2) and threshold (*τ* = 0.35), with the same number of drugs (*d* = 8), and choosing their inhibition targets in a random manner (unfeasible problems are ignored). However, since on very large networks it is quite unlikely to induce the sought modulation on the objective reaction when only 8 targets are inhibited, the number of targets of each drug is proportionally increased (on average the total number of inhibitions is approximately 6% of the total number of reactions). Because of the randomness in the choice of the targets, the computational times may present a significant variation. Therefore, Figure 3 (b) shows the whole distributions of the computational time over 100 runs for each one of the six metabolic networks we considered. Finally, Figure 3 (c) reports the mean and the standard deviation of these distributions as a function of the size of the network. On average, also for very large networks, the computational time is approximately one hour (on a 2.3 GHz CPU). All these characterizations show the good performaces of the algorithm.

### Screening for optimal drug combinations

**Figure 4:**
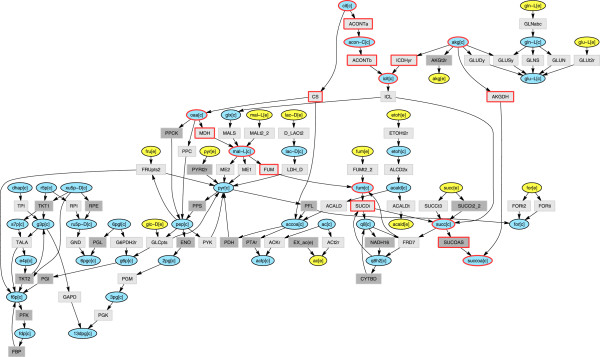
**Network representation of** ***E. coli* core metabolism.** This reconstruction refers to the work of Orth *et al.*[35] and maintains the same notation. Metabolites are in ellipses (yellow color: external nutrients; blue color: cytoplasmatic compounds) and reactions in boxes (dark gray for irreversible and light gray for reversible processes). Nodes highlighted with red border belong to Krebs cycle. For a better readability, common species like water, Oxygen, H^+^, Ammonium, CO_2_, phosphate, NAD (in all forms), Coenzyme-A, AMP, ADP and ATP, as well as all transport and exchange processes, have been excluded from the representation.

The main scope of these calculations is to show the advantage given by the use of value of *P* higher than zero, i.e. of passing from the ON/OFF to a more accurate description. In order to better characterize its behavior (performing a large number of tests), we run the algorithm on the small network of the core metabolism of *E.coli*. A sketch of this network is depicted in Figure 4.

A set of tests have been carried out combining different values of *τ* and *P*, in particular:

$$\begin{array}{l} \tau \in \{0.0,\;0.1,\;0.5,\;1.5,\;2.0\};\\ P\in \{0,\;1,\;2\}. \end{array}$$

For each pair, we perform a screening that considers each metabolic reaction as objective process to be modulated (down- or up-regulated depending on the value of *τ*) and finds the most selective drug combination. The following characterization of the solutions is performed. For a given *P* and for a given objective reaction *v*_mod_, we consider the solutions **h** at different values of *τ*. When the same drug combination is found for two values of *τ* (for example *τ*_1_ = 0.1 and *τ*_2_ = 0.5), the solution is considered valid only for the most stringent constraint (*τ*_1_ = 0.1, in the example; similarly, if *τ*_1_ = 1.5 and *τ*_2_ = 2.0 then the solution is associated to *τ*_2_ = 2.0 only). This procedure allows to considered only cases when passing to a weaker constraint on *v*_*mod*_ the severity (for instance the dosage) of the corresponding optimal drug treatment is reduced too. We analyze the results by looking at the following four indices.

#### 

**Figure 5:**
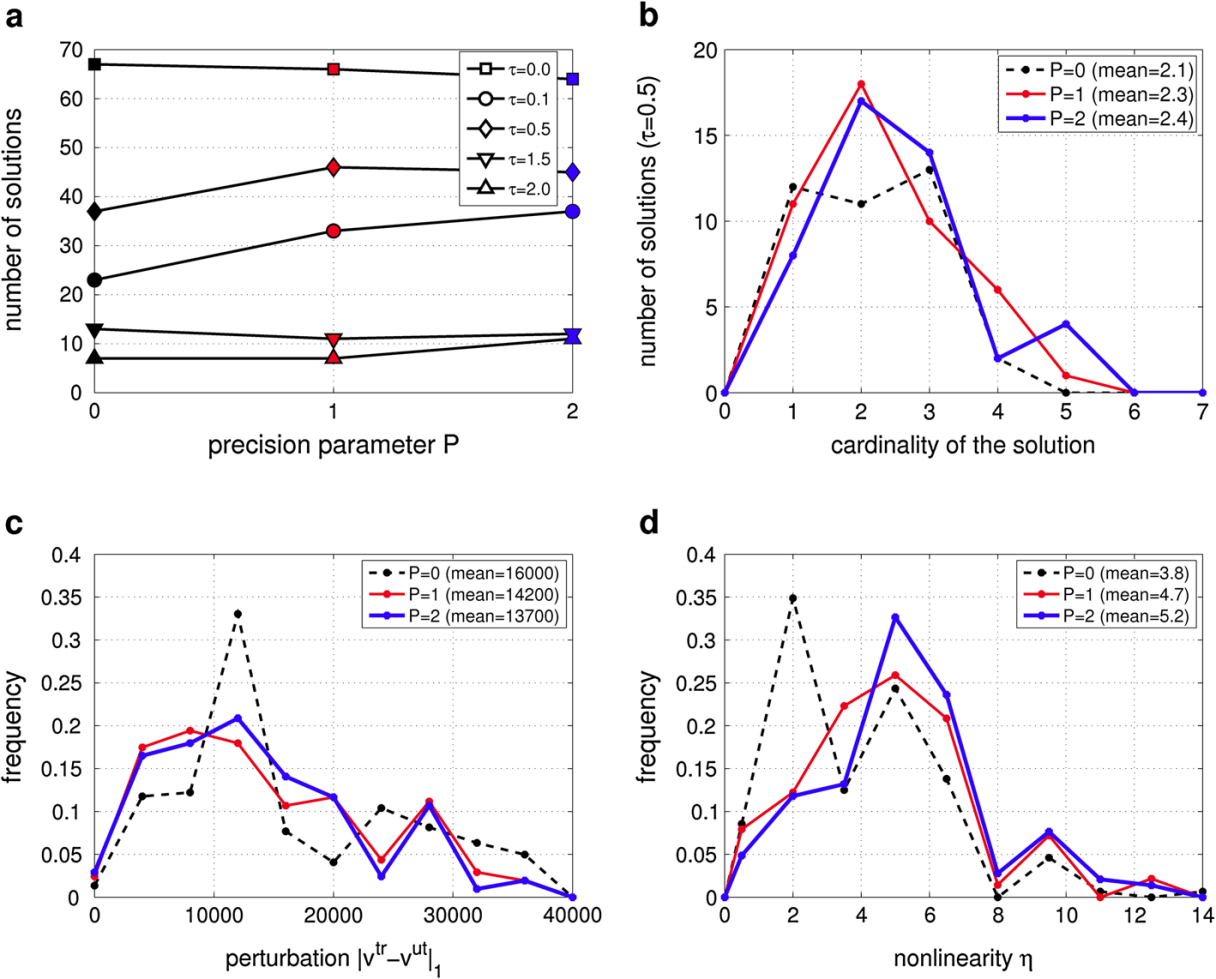
**Effect of the precision parameter** ***P* (results from the reactions screening).** **(a)** The plots show the number of solutions that are found for different values of *τ* (see legend) as function of *P* (color codes as in the following plots). As one can see, for *τ* > 0, increasing the precision *P*, it is nearly always more frequent to find a drug combination which induces the sought modulation. **(b)** The plot is a detail of the curve in panel (a) for the case *τ* = 0.5, and shows the frequency of the drug cardinality of the solutions (i.e. the number of drugs used in the solution). For larger *P*, the distribution is slightly shifted to higher cardinality (data for each *τ* are reported in Table 3). **(c)** Beside the number of solutions, higher precision produces more selective outcomes, i.e. with a lower side effect (as clearly shown by the mean values reported in the legend). The counting is performed over all different values of the threshold *τ*. **(d)** This plot shows the histogram of the values of the nonlinearity index *η*(**h**) (as reported in (11)) calculated for all the solutions of the screening (still regardless the value of *τ*). From the curves and from the mean values reported in the legend, it is possible to see that higher amount of nonlinearity are obtained when *P* is increased.

**Number of solutions:** Figure 5 (a) shows the total number of solutions we have found in the screening of all reactions at different *P* and *τ*. One can see that, when a complete stop of the objective reaction is required (*τ* = 0) there is no significant advantage in increasing the precision *P*. However, when it is necessary to induce a more accurate modulation of the flux (inhibitory when 0 < *τ* < 1), higher values of *P* allow to find a larger number of solutions. Through the partial inhibition, indeed, we can find solutions which are closer to the desired threshold, whereas the simple ON/OFF model can mostly induce a complete stop of the objective reaction. A similar improvement can be also identified while passing from *τ* = 1.5 to *τ* = 2.0.

**Table 3:** Averaged cardinality of the drug combinations from the screening

	***τ*** **= 0.0**	***τ*** **= 0.1**	***τ*** **= 0.5**	***τ*** **= 1.5**	***τ*** **= 2.0**
*P* = 0	2.5	2.9	2.1	2.1	1.4
*P* = 1	2.7	3.0	2.3	3.5	3.1
*P* = 2	2.9	3.3	2.4	3.7	3.2

Figure 5(b) shows the details of the screening results for *τ* = 0.5 and the corresponding legend reports the averaged value of the cardinality of the solutions which have been found; we summarize here the same quantities for all values of *τ*.

#### 

**Cardinality of the solutions:** More details are presented in panel (b) of Figure 5, which reports the histogram of the cardinality of the solutions and their mean values for the case of *τ* = 0.5 (averages for each value of *τ* are reported in Table 3). When the precision increases, the distribution of the cardinality shifts slightly to higher values, meaning that multiple drug treatments are (slightly) preferred.

#### 

**Perturbation induced by the solutions:** For each solution that we have identified during this screening, also the corresponding perturbation (i.e. the side effect ∥**v**^tr^-**v**^ut^∥_1_) can be evaluated. We calculate the frequency of these perturbation values (regardless of the value of *τ*). The result is shown in Figure 5 (c). We notice that at higher precision, smaller perturbations become slightly more probable: as expected, for high values of *P*, the algorithm can modulate the inhibition more accurately and therefore reduce the impact on the network, while still satisfying the request on the flux of the objective reaction.

#### 

**Nonlinearity exploited by the solutions:** The interaction between drugs is normally interpreted as the deviation of the effect of combined drugs with respect to the linear superposition of the single drug perturbations. Therefore, similarly to the scaled epistasis measure presented in literature [42], a index of nonlinearity *η*(**h**) can be defined on the basis of the flux of the objective reaction as follows. Let $v_{\text{mod}}^{\text{tr}}(h_{1},h_{2},\ldots ,h_{d})$ be the flux of the objective reaction at drug inhibition **h** = (*h*_1_,*h*_2_,…,*h*_*d*_) and given by (5). Then,

(11) $$\eta (h_{1},\ldots ,h_{d}):=\frac{v_{\text{mod}}^{\text{tr}}(h_{1},0,\ldots ,0)+\cdots +v_{\text{mod}}^{\text{tr}}(0,\ldots ,0,h_{d})-(d-1)v_{\text{mod}}^{\text{ut}}-v_{\text{mod}}^{\text{tr}}(h_{1},\mathrm{...},h_{d})}{v_{\text{mod}}^{\text{ut}}-v_{\text{mod}}^{\text{tr}}(h_{1},\ldots ,h_{d})},$$

since it holds $v_{\text{mod}}^{\text{ut}}=v_{\text{mod}}^{\text{tr}}(0,0,\ldots ,0)$. From this definition, *η* = 0 means linear behavior and *η* > 0 nonlinear. Therefore, for each solutions of the screening, we calculate the corresponding *η*(**h**) and we analyzed the distribution of its values (still ignoring the parameter *τ*): the result is shown in panel (d) of Figure 5. It is clear that increasing the value of *P* the nonlinearity index tends to be higher. It seems that, thanks to the higher precision, the algorithm may exploit more efficiently the nonlinearity property and, by consequence, it can limit the dosage of the drug and consequently reduce the perturbation.

### Drug interaction surfaces: three case studies

**Figure 6:**
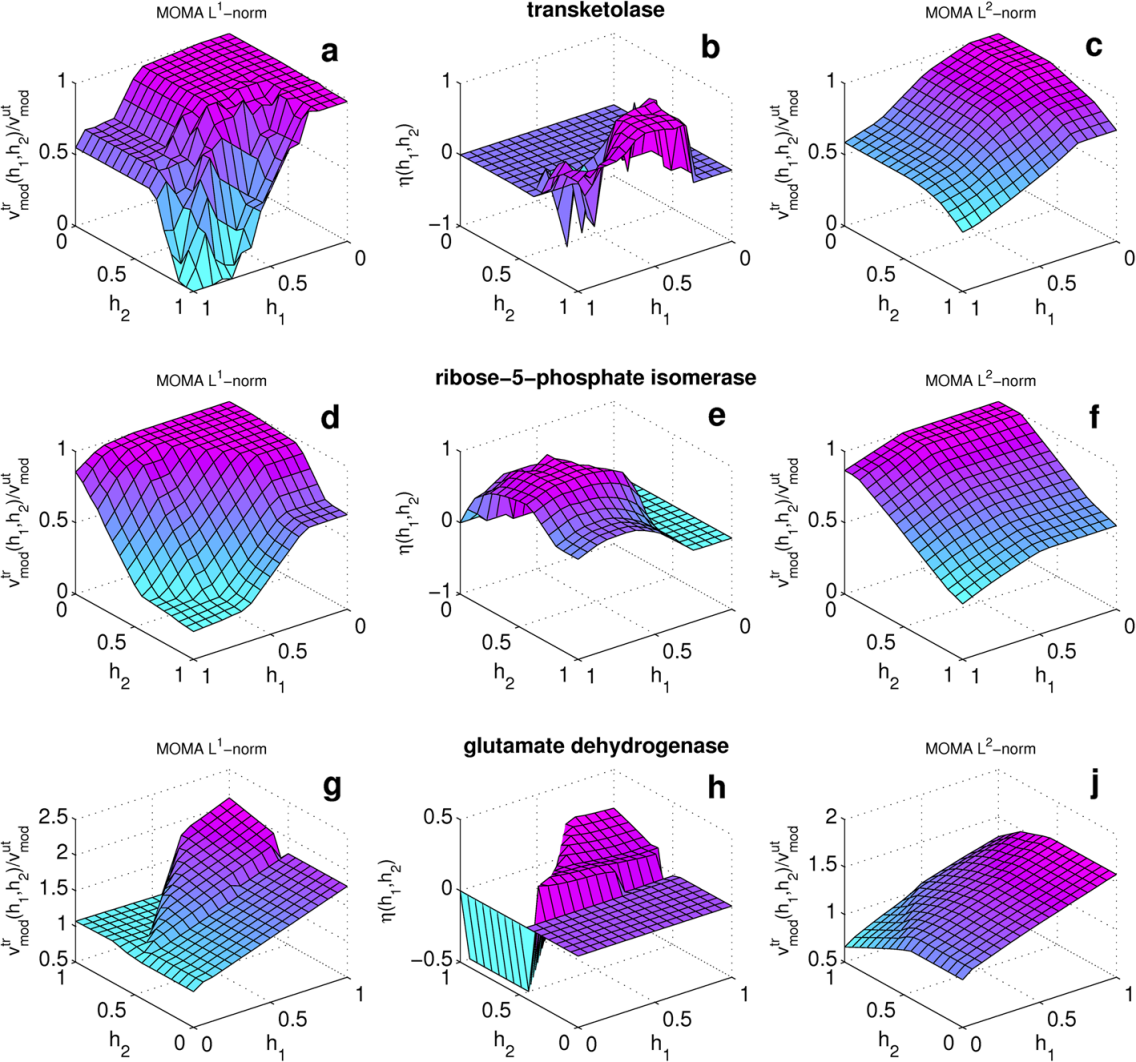
**Surfaces of drug interaction.** Axes *x* and *y* report the inhibition coefficients *h* (dosage) for the two drugs; the *z*-axis reports the percentage of the flux through the modulated reaction after drug treatment calculated with *L*^1^ formulation of MOMA with respect to the untreated value (left panels **a**, **d** and **g**), or the relative deviation, *η*(**h**), of the effect induced by the drug combination from the linear superposition of the effects due to the single drugs as expressed by (11) (middle column, panels **b**, **e** and **h**). The right panels (**c**, **f** and **j**) show the calculations made with MOMA based on the *L*^2^-norm. The higher smoothness of the surface makes this formulation more reliable: however the surfaces obtained with our method (*L*^1^-norm) well reproduce the main characteristic of the synergism between the drugs. In the first two rows inhibitory synergisms are shown (*τ* < 1), whereas the third row is an example of activating synergism (*τ* > 1). Notice that for the sake of readability, in the plots of the last row, both *x* and *y* axes have been inverted.

For three of the solutions found through the screening procedure, we detail now the drug interactions exploited by the algorithm. In particular, we considered the synergisms in the inhibition of *transketolase* and of *ribose-5-phosphate isomerase*, and the synergism in the up-regulation of *glutamate dehydrogenase*. Each of the first two solutions contains a pair of drugs (Fomepizole plus Halofantrine and Fomepizole plus Hexachlorophene respectively). We explore the drug interaction surface changing the amount of inhibition induced by each compound, as could correspond in experiments to using different drug dosages (the interval [0,1] has been discretized using (9) with *P* = 4). The 2D surfaces are reported in Figure 6 panels (a) and (d). In the third case (up-regulation of *glutamate dehydrogenase*, panel (g)) the synergism is obtained combining three drugs (Nitrofurazone, Halofantrine and Pemetrexen); therefore, in order to have the 2D surface of interaction, the first drug is kept at the optimal inhibition value (*h*_1_ = 1) and the combinations are explored changing the dosages of the remaining two drugs. Figure 6 reports also the nonlinearity index *η*(**h**) as defined in (11); panels (b), (e) and (h) show that in all cases there is a clear enhancement of the effect when the drugs are combined.

These calculations has been performed with MOMA based on the *L*^1^-norm because, as already mentioned, it allows a definition of a linear function in the optimization problem. When compared to the surfaces obtained from the original quadratic formulation of MOMA (last column: panels (c), (f) and (j) of Figure 6), the results from *L*^1^-norm show some irregularity of the surfaces (which makes the original *L*^2^ version more reliable) but the main features of the drug-drug interaction are still well described.

## Discussion

Optimization is a concept widely used in many scientific fields; for instance, in systems biology, FBA makes use of it for discriminating reaction fluxes in large metabolic networks. Following the same philosophy, in order to cope with more complex situations, multiple optimization criteria can be needed simultaneously leading in some situations, like the one discussed in this paper, to a bilevel optimization problem. The bilevel approach is promising for studying several features and applications of metabolic networks, for instance for identifying metabolic objective functions [23] or for studying perturbations around a nominal optimum [10,11]. In the context of drug combinatorics, in order to efficiently solve the bilevel optimization, Boolean variables are commonly used in the outer problem. However, this ON/OFF description of the corresponding biological quantities may represent a very rough approximation, as it is the case for the (partial) inhibition induced by drugs acting on the enzymes of a metabolic network.

In order to overcome this limitation, we propose an improvement on the formulation of the bilevel optimization in which a single Boolean variable is replaced by a convex combination of several Boolean quantities: in this manner the convex and linear nature of the problem is preserved and the description of the inhibitory effects becomes more realistic. Since the problem contains Boolean variables, the optimization falls in the Mixed Interger Linear Programming (MILP) class: compared to LP, the *NP*-hard complexity of MILP [22] makes the new algorithm more expensive from a computational point of view. For the tasks at hand (see Figure 3), the algorithm behaves well also for large metabolic networks. The logarithm of the computational time scales linearly with the number of reactions, but with a small slope, so that on average the solution is found in a reasonable computational time, also for networks with around 2500 reactions and for *P* = 2.

For testing purposes, we run the algorithm on the central carbon metabolism of *E.coli* screening all reactions. We have found that increasing the number of Boolean variables used in the convex combination (the precision parameter *P*), it is more likely to find a solution which succeeds on the modulation of the objective reaction (see Figure 5 (a)). In particular, partial inhibitions (i.e. modulations of the dosage of the drugs) are more frequent for multicomponent solutions (panel (b) of Figure 5): this result may be interpreted as a wider possibility, offered by the synergism, to calibrate a drug treatment according to the specific needs. Moreover our computations represent a confirmation on large networks of the expected, but still not verified, higher efficiency of multiple targets drug treatments in presence of partial inhibition [24]. In this perspective, the results show that this approach may also lead to treatments which are more selective (panel (c) of Figure 5 and Table 3).

A possible explanation can be found in the unexpected or hardly predictable drug synergism which are typical of complex systems such as metabolic networks, even in a simplified framework like FBA. In particular, concerning the synergistic interactions between drugs, the analysis done through the drug-drug interaction surface (Figure 6) reveals that nonlinear effects, not explained by superposition of the single drug perturbation, are significant and can be captured and exploited by the method proposed, unlike with a more coarse-grained ON/OFF description. We should mention, that the three case studies presented in Figure 6 do not pretend to have any clinical value: they have been selected only for the purpose of illustrating the method and the advantages it may give in the context of drug synergism and drug reprofiling for reconstructed metabolic networks.

## Conclusion

The purpose of this paper is to present a novel algorithm, able to relax the assumption on the variables of a bilevel optimization problem from ON/OFF type to more fine-graded description. This setting is of interest in the context of FBA of metabolic networks and in particular in the modulation of the fluxes by means of drugs, capable of reducing (but not suppressing completely) the activity of the metabolic enzymes on which they are acting. With our algorithm, the problem can be formulated as a MILP problem of moderate practical complexity. Indeed we have shown that the algorithm performs well also on large metabolic networks at a more fine-graded level of resolution than an ON/OFF representation. Furthermore, it is capable of exploiting with higher efficiency the peculiar nonlinearity which originates from the topology of the network, of finding more selective solutions and, therefore, of offering a larger spectrum of drug combinations. These features become more evident when the modulation required for the objective reaction is itself a fraction (*τ* ≠ 0) of the nominal flux, rather than a simple complete switch off (*τ* = 0). Indeed, if a disease corresponds to an anomalous biosynthesis of certain compounds, most often the cure consists in regulating back those fluxes to an healthy range, not to a complete inhibition.

It is worth noting that the problem of drug synergism we presented in this paper must be read as a guiding example for a more general class of situations: indeed, the idea we have proposed for treating bilevel optimization can be applied to any other case which requires a more realistic modeling with respect to the oversimplified ON/OFF description, in biology as well as in all the other fields where LP is already used.

## Abbreviations

FBA: Flux balance analysis; MOMA: Minimization of metabolic adjustment; LP: Linear programming; MILP: Mixed integer linear programming.

## Competing interests

The authors declare that they have no competing interests.

## Authors’ contributions

GF developed the method and performed the calculations. CA and GF participated at the discussions of the results, have been involved in drafting the manuscript and approved the final manuscript’s preparation.

## Supplementary Material

Additional file 1 **Nonuniquess.** This pdf file presents the behaviour of the algorithm in case of nonunique solution of the untreated network (**v**^ut^). Click here for file

Additional file 2 **Matrix of linear constraints.** This pdf file provides the details about the construction of the matrix of the final single optmiziation problem, including primal, dual and Boolean variables of the drugs. Click here for file
