# Supplementary material for: Partial inhibition and bilevel optimization in flux balance analysis
Source: BMC Bioinformatics. 2013 Nov 29;14:344. doi: 10.1186/1471-2105-14-344 (PMC4219332; doi:10.1186/1471-2105-14-344)
Supplement: Additional file 1 — Nonuniquess. This pdf file presents the behaviour of the algorithm in case of nonunique solution of the untreated network (vut). [file 1471-2105-14-344-S1.pdf]

Partial inhibition in FBA through a bilevel optimization

		Variabiles of INNER PROBLEM								Variables of OUTER PROBLEM			Left hand term
		primal variables		duals variables associated to "..."						P=2			
		fluxes	MOMA	"steady state"	"upper bound"	"absolute values"		"inhibition"	"nonlinearity equivalences"	Drug h_1	Drug h_1	Drug h_d	
		v	a	mu	lambda	alpha	beta	delta	z	x11 x12 x13	x21 x22 x23	x31 x32 x33	
	size	r	r	m	r	r	r	t	t*d	P	P	P	1
Primal problem<br>Sv=0	m	S											0<br>0<br>0<br>0
duality theorem equality	1		-1 -1 -1 -1 -1 -1	0 0 0 0	-U -U -U -U -U -U	-v^ut -v^ut -v^ut -v^ut -v^ut -v^ut	-v^ut -v^ut -v^ut -v^ut -v^ut -v^ut		-U*b				0
v<=U	r	1<br>1<br>1<br>1<br>1<br>1											U<br>U<br>U<br>U<br>U<br>U
absolute value<br>-a+v<=+v*	r	1<br>1<br>1<br>1<br>1<br>1	-1<br>-1<br>-1<br>-1<br>-1<br>-1										+v*<br>+v*<br>+v*<br>+v*<br>+v*<br>+v*
-a-v<=-v*	r	-1<br>-1<br>-1<br>-1<br>-1<br>-1	-1<br>-1<br>-1<br>-1<br>-1<br>-1										-v*<br>-v*<br>-v*<br>-v*<br>-v*<br>-v*
V-Ud<=0	t	1<br>Di 1<br>1								Ub1 Ub2 Ub3	Ti<br>Ub1 Ub2 Ub3	Ub1 Ub2 Ub3	0<br>0<br>0
dual problem	r			Transpose<br>-S	-1<br>-1<br>-1<br>-1<br>-1<br>-1	-1<br>-1<br>-1<br>-1<br>-1<br>-1	1<br>1<br>1<br>1<br>1<br>1	-1<br>Transpose<br>-Di<br>-1<br>-1					0<br>0<br>0<br>0<br>0<br>0
	r					1<br>1<br>1<br>1<br>1<br>1	1<br>1<br>1<br>1<br>1<br>1						1<br>1<br>1<br>1<br>1<br>1
non linearity<br>z-Dd<=0	t*d								1<br>1<br>1<br>1<br>1<br>1<br>1<br>1<br>1	-delta^max<br>-delta^max<br>-delta^max	-delta^max<br>-delta^max<br>-delta^max	-delta^max<br>-delta^max<br>-delta^max	0<br>0<br>0<br>0<br>0<br>0<br>0<br>0<br>0
z-delta<=0	t*d							-1<br>-1<br>-1<br>-1<br>-1<br>-1<br>-1<br>-1<br>-1	1<br>1<br>1<br>1<br>1<br>1<br>1<br>1<br>1				0<br>0<br>0<br>0<br>0<br>0<br>0<br>0<br>0
-z+delta+Dd<=D	t*d							1<br>1<br>1<br>1<br>1<br>1<br>1<br>1<br>1	-1<br>-1<br>-1<br>-1<br>-1<br>-1<br>-1<br>-1<br>-1	delta^max<br>delta^max<br>delta^max	delta^max<br>delta^max<br>delta^max	delta^max<br>delta^max<br>delta^max	delta^max<br>delta^max<br>delta^max<br>delta^max<br>delta^max<br>delta^max<br>delta^max<br>delta^max<br>delta^max
v_mod<=tau*v_mod^ut	1	Index mod=1											tau*v_mod^ut
Outer problem Functional			1 1 1 1 1 1							-ab1 -ab2 -ab3	-ab1 -ab2 -ab3	-ab1 -ab2 -ab3	
