# Supplementary material for: Partial inhibition and bilevel optimization in flux balance analysis
Source: BMC Bioinformatics. 2013 Nov 29;14:344. doi: 10.1186/1471-2105-14-344 (PMC4219332; doi:10.1186/1471-2105-14-344)
Supplement: Additional file 2 — Matrix of linear constraints. This pdf file provides the details about the construction of the matrix of the final single optmiziation problem, including primal, dual and Boolean variables of the drugs. [file 1471-2105-14-344-S2.pdf]

Partial inhibition and bilevel optimization in Flux Balance Analysis SUPPLEMENTARY INFORMATION

Giuseppe Facchetti and Claudio Altafini

Exploring alternative unperturbed fluxes $\mathbf{v}^{\mathrm{ut}}$

As mentioned in the main text of the paper, the optimization of the biomass production may lead to a nonunique solution $\mathbf{v}^{\mathrm{ut}}$. We presume that the nonuniqueness is mainly due to the presence of circulations and alternative equivalent pathways in the network, a problem which is well known in FBA and which is not ascribable to our algorithm. Nevertheless, in this Supplementary Notes we analyze the behavior of the algorithm with respect to the variability of the unperturbed fluxes.

Procedure: The set $M \subset W$ of solutions at maximal growth rate $v_{\mathrm{biomass}}^{\mathrm{max}}$ can be defined jointly by the standard FBA constraints and by the extra equality constraint on v_{biomass}:

$$\begin{aligned} \mathbf{S}\mathbf{v} &= \mathbf{0} \\ 0 \leq v_i &\leq U_i \qquad \forall i = 1, \ldots, r \\ v_{\mathrm{biomass}} &= v_{\mathrm{biomass}}^{\mathrm{max}}. \end{aligned}$$

In order to perform a robustness analysis of the algorithm, we sample points in M and apply the following procedure:

1) We choose *ribose-5-phosphate isomerase* as objective reaction of the *E.coli* core metabolic network and we set $P = 2$, $\tau = 0.6$.

2) The FBA solution with optimal biomass production is denoted by $\mathbf{v}^{\mathrm{ut}}(0)$ (therefore we have $v_{\mathrm{biomass}}^{\mathrm{max}} = v_{\mathrm{biomass}}(0)$) whereas $\mathbf{v}^{\mathrm{tr}}(0)$ is the corresponding solution of the algorithm (treated metabolic fluxes). The counter n is set equal to 0.

3) The counter n is increased. Starting from $\mathbf{v}^{\mathrm{ut}}(n-1)$ we randomly choose a new vector of fluxes which has the same maximal growth rate (in practice we do a random walk in M). This is achieved by a adding Gaussian random vector to $\mathbf{v}^{\mathrm{ut}}(n-1)$ and projecting the new point on M (the projection of a Gaussian random vector on a hyperplane is still Gaussian). This generates $\mathbf{v}^{\mathrm{ut}}(n)$.

4) We run the algorithm using $\mathbf{v}^{\mathrm{ut}}(n)$ and obtaining the corresponding $\mathbf{v}^{\mathrm{tr}}(n)$.

5) Points 3) and 4) are repeated iteratively for 100 times.

In the end we have two set of vectors: $\{\mathbf{v}^{\rm ut}(j)\}_{j=0,\dots,100}$ and the corresponding $\{\mathbf{v}^{\rm tr}(j)\}_{j=0,\dots,100}$. For each pair (j,k), $j=0,\dots,100$, $k>j$, we calculate the normalized distances:

$$d^{\rm ut}_{j,k} = \frac{||\mathbf{v}^{\rm ut}(j)-\mathbf{v}^{\rm ut}(k)||_1}{||\mathbf{v}^{\rm ut}(0)||_1},$$

$$d^{\rm tr}_{j,k} = \frac{||\mathbf{v}^{\rm tr}(j)-\mathbf{v}^{\rm tr}(k)||_1}{||\mathbf{v}^{\rm ut}(0)||_1}.$$

Both normalization are performed with respect to the same quantity $||\mathbf{v}^{\rm ut}(0)||_1$.

Results: Results are reported in Figure S1. Panel A shows that there is certain degree of correlation between the two distances.
The histogram analysis of the ratio between them (i.e. $d^{\rm tr}_{j,k}/d^{\rm ut}_{j,k}$) is presented in panel B. Since this ratio is always smaller than 1, these results indicate that starting from different $\mathbf{v}^{ut}$, the deviation among the solutions of the algorithm is smaller that the variation of the $\mathbf{v}^{ut}$, meaning that the algorithm reduces (or at least it does not amplify) the variability of the starting point.

This can be justified by the fact that the algorithm is a "contraction", i.e. , denoting by $\mathcal{F}$ the map corresponding to the algorithm, for any pair of vectors $\mathbf{v}$ and $\mathbf{w}$, the following inequality holds:

$$||\mathcal{F}(\mathbf{v})-\mathcal{F}(\mathbf{w})||_1 \le ||\mathbf{v}-\mathbf{w}||_1.$$

Indeed, the algorithm starts with the unperturbed convex set W of feasible fluxes defined by the standard FBA constraints. Then, this set is reduced to a proper convex subset $W(\mathbf{h})$ through the inhibition induced by the drugs (selected according to the modulation of the objective reaction and to the side effect). Finally, MOMA performs a projection of the unperturbed fluxes $\mathbf{v}^{\rm ut}$ on this subset. Therefore, as for any projection on a convex set in a metric space (the metric being induced by the L^1 norm), the algorithm reduces distances.

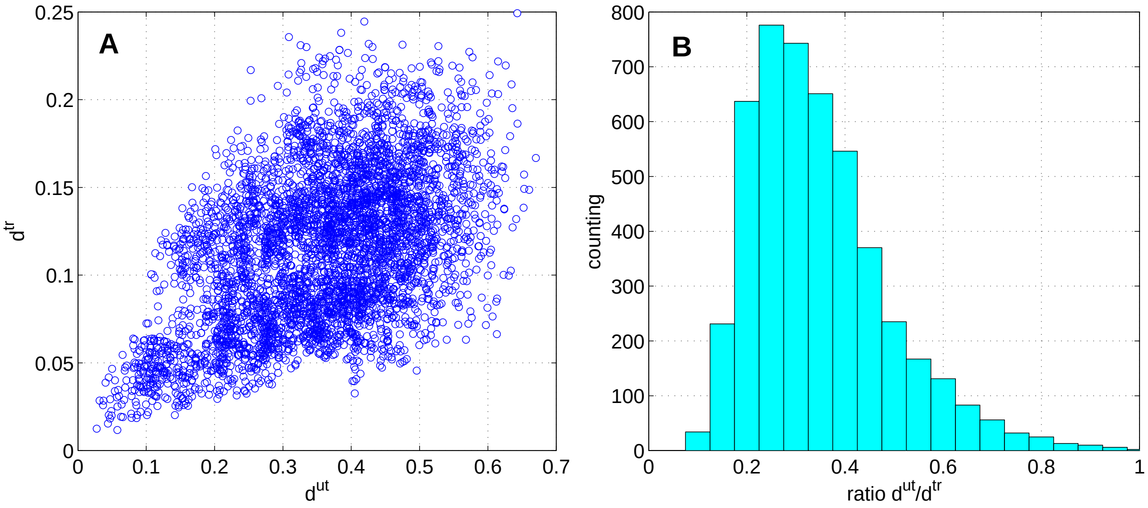

Figure S1: Results from 100 different unperturbed fluxes $v^{\rm ut}$ (4950 pairs) at the same biomass production.
Panel A: Correlation between $d^{\rm ut}_{j,k}$ and $d^{\rm tr}_{j,k}$. *Panel B:* Distribution of the ratio $d^{\rm tr}_{j,k}/d^{\rm ut}_{j,k}$.
